# Preclinical and clinical evidence on the approach-avoidance conflict evaluation as an integrative tool for psychopathology

**DOI:** 10.1017/S2045796022000725

**Published:** 2022-12-13

**Authors:** F. Rusconi, M. G. Rossetti, C. Forastieri, V. Tritto, M. Bellani, E. Battaglioli

**Affiliations:** 1 Department of Medical Biotechnology and Translational Medicine, Università degli Studi di Milano, Milano, Italy; 2 Department of Neurosciences and Mental Health, Fondazione IRCCS Ca’ Granda Ospedale Maggiore Policlinico, Milan, Italy; 3 Department of Neurosciences, Biomedicine and Movement Sciences, Section of Psychiatry, University of Verona, Verona, Italy

**Keywords:** Biological markers, brain imaging techniques, diagnosis and classification, psychological assessment

## Abstract

The approach-avoidance conflict (AAC), i.e. the competing tendencies to undertake goal-directed actions or to withdraw from everyday life challenges, stands at the basis of humans' existence defining behavioural and personality domains. Gray's Reinforcement Sensitivity Theory posits that a stable bias toward approach or avoidance represents a psychopathological trait associated with excessive sensitivity to reward or punishment. Optogenetic studies in rodents and imaging studies in humans associated with cross-species AAC paradigms granted new emphasis to the hippocampus as a hub of behavioural inhibition. For instance, recent functional neuroimaging studies show that functional brain activity in the human hippocampus correlates with threat perception and seems to underlie passive avoidance. Therefore, our commentary aims to (i) discuss the inhibitory role of the hippocampus in approach-related behaviours and (ii) promote the integration of functional neuroimaging with cross-species AAC paradigms as a means of diagnostic, therapeutic, follow up and prognosis refinement in psychiatric populations.

Decision-making results from a complex system of corticolimbic brain structures: the *mesolimbic pathway* is responsible for encoding motivation and promoting actions (Goto and Grace, 2005), while the hippocampus, the amygdala and the prefrontal cortex (PFC) assign an affective value to life experiences (Calhoon and Tye, 2015). These areas integrate the dopaminergic activity of the mesolimbic pathway, ultimately promoting or inhibiting the action (Adhikari *et al*., 2010; Abela *et al*., 2013; Abela and Chudasama, 2014). Thus, a healthy individual undertakes goal-directed actions as a result of an *inner conflict* among corticolimbic brain areas, which ultimately drives the behavioural choice to approach or avoid a life paradigm, following a continuous gradient of integration between needs, rewards and risks (Cornwell *et al*., 2014). This ‘conflict’ is usually referred to as the approach-avoidance conflict (AAC) and its disbalance, in both directions, represents a highly shared symptom of several psychiatric disorders (Aupperle and Paulus, 2010; Aupperle *et al*., 2015; Kirlic *et al*., 2017; Loijen *et al*., 2020). According to Gray's Reinforcement Sensitivity Theory (RST), approach-avoidance behaviour arises from a balanced interaction between the Behavioural Approach System (BAS) and the Behavioural Inhibition System (BIS) (Gray, 1970; Gray and McNaughton, 2003; Bijttebier *et al*., 2009). In mammals, BAS promotes behavioural responses to appetitive and rewarding stimuli, while BIS organises individuals' responses to punishing or threatening situations (Gray and McNaughton, 2003). BIS and BAS are informed by implicit and explicit information processing, involved in positive or negative valence attribution to specific cues (Loijen *et al*., 2020). Implicit information processing is fast and emotion-driven and depends on subcortical brain regions involved in affective processing (i.e. mesolimbic areas including the hippocampus and amygdala). Conversely, explicit information processing is relatively slow, requires intentional reasoning and attentional efforts, and seems to depend on frontal brain regions associated with cognitive control and emotional reappraisal (Wager *et al*., 2008; Aupperle and Paulus, 2010). Gray's RST assumes that individuals with an unbalanced BIS/BAS ratio are at risk of psychiatric drift (Loijen *et al*., 2020). Consistently, dysfunctional approach-avoidance tendencies have been implicated in the development and progression of several mental health disorders such as anxiety, depression, eating and addictive disorders and schizophrenia (Bijttebier *et al*., 2009; Struijs *et al*., 2017; Loijen *et al*., 2020). We aim to emphasise the translational role of AAC paradigms in clinical psychiatric research, by discussing preclinical and clinical evidence of the brain circuits associated with AAC.

## Cross-species paradigms to assess AAC

Cross-species behavioural tests aim at resolving shared molecular and circuital determinants between humans and rodents that underlie homologous disease-relevant behaviours in the AAC domain (Bach, 2021). This approach should build a more solid basis to develop novel tools to help clinical diagnosis and maximise the *predictive validity* of AAC preclinical paradigms used to test pharmacological interventions for humans (Belzung and Lemoine, 2011). Cross-species behavioural tests to assess AAC mainly exploit paradigms of exploration (Gromer *et al*., 2021). Mammals' functioning is indeed based on an innate exploratory drive for territorial recognition, food supply and reproduction, which are in turn associated with a significant reward (Kidd and Hayden, 2015). However, exploration naturally entails a conflict between its reward component, and the objective risk (Arzate-Mejía *et al*., 2020; Italia *et al*., 2020). Thus, explorative engagement results from a fine balance between motivation (driven by reward) and refusal (supported by fear) (Blanco *et al*., 2013; La-Vu *et al*., 2020). Exploratory avoidance (which also includes to some extent social avoidance) is a pathological status that can either be caused by a loss of the natural desire to explore and socialise (anhedonia) or can be the result of a pathological fear (anxiety) (Kim and Kirkpatrick, 1996; Arzate-Mejía *et al*., 2020). The first case is more evocative of a depressive-like drift, the second is associated with an aberrant state of anxiety which prevents an individual from indulging in social and exploratory needs, even though social and exploratory desire can be intact (Bijttebier *et al*., 2009). In the following sections, we describe those AAC paradigms that are suitable for both humans and rodents.

In rodents, one of the most used exploratory paradigms to assess AAC is the *Open Field* (OF) test. It consists of a simple observation of animal deambulatory behaviour (Prut and Belzung, 2003). Modern software digitally traces the distance moved by the rodent from the perimetric walls to the centre of an arena. The longer the time spent and the distance walked in the centre, the more a rodent is prone to ‘approach’ exploration (Noldus *et al*., 2001). Conversely, animals walking near the perimetric walls of the cage (also known as thigmotaxis) adopt an instinctive behaviour aimed at protecting against a perceived novelty-related threat (Prut and Belzung, 2003). In literature, the OF test is almost often described as an *anxiety-assessing* paradigm. However, it is important to underline that decreased exploration is also due to decreased exploration-related reward sensitivity (Blanco *et al*., 2013; Cornwell *et al*., 2014; La-Vu *et al*., 2020).

Human versions of the OF test have been developed and administered to clinical/non-clinical groups. Kallai *et al*., measured human thigmotaxis during the exploration of virtual and physical spaces, showing that thigmotaxis positively correlated with fear and avoidance bias for fear-mobilising situations during early trials of both tasks, but not with self-reported trait anxiety (Kallai *et al*., 2007). Walz *et al*., instructed patients with agoraphobia and healthy controls to perform a 15 min solitary walk on a 146 × 79 m soccer field. Patients with agoraphobia and participants with high self-reported anxiety sensitivity exhibited enhanced thigmotaxis (Walz *et al*., 2016). In an additional virtual reality OF test performed on 141 individuals, the participants – like rodents in animal studies – preferred to stay in the outer region of the open field but there was no consistent association between thigmotaxis and self-report scales of anxiety and fear (Gromer *et al*., 2021). Overall, human OF test demonstrated cross-species validity, although, the modulatory effects of anxiety on human open-field behaviour should be further examined (Bach, 2021).

Another exploratory paradigm frequently used in rodents is the *elevated plus maze* (EPM) test (Walf and Frye, 2007). The maze, a cross-like structure mounted on elevated strut, is made up of two *open* and two *closed arms* where opaque plexiglass walls protect the rodent path (Rusconi *et al*., 2016). Closed arms are more comfortable for rodents and represent the preferred part of the maze. However, instinctive propensity for exploration prompts the animals to abandon the closed arms for the open ones for a short time. The percentage of time spent and the frequency of open arms entries represent a reliable readout of the rodents' propensity to approach. EPM test is largely used as an anxiety-assessment test that, however, does not take into consideration the reward-related drive to explore. Thus, these two tests specifically measure the AAC, as the result of the contribution of two components: anxiety and reward sensitivity (Cornwell *et al*., 2014; Bryant and Barker, 2020).

In humans, the EPM corresponding paradigm is the *Mixed Reality version of EPM*. Biedermann *et al*. (2017) translated the rodent EPM test to humans using a combination of real-world and virtual elements namely, a real-world wooden maze combined with a representation of this maze in virtual reality. Briefly, participants were instructed to step into the maze and walk slowly towards its centre, and wait for the scene to change before exploring the environment of the maze. After 90 s, the scenario switched and, instead of being in a virtual room, the maze was placed on a virtual rocky mountain surrounded by water. The subjects were allowed to explore the EPM for 300 s and, reporting higher anxiety about open arms, they preferentially avoided them. This tendency increased or decreased when they were given the anxiogenic yohimbine and the benzodiazepine lorazepam, respectively.

Other cross-species approaches featuring operant conflict tests were developed to emphasise both anxiety and reward sensitivity components thus further enhancing the conflict load of the choice (Bach, 2021). These tests are based on the association of a specific reward or punishment to a given action. In rodents, the *Vogel conflict* test (VCT) represents one of the best constructs of AAC (Millan and Brocco, 2003). In this paradigm, within a habituation phase, a thirsty animal learns to drink from a metallic gauge. During the trial phase, after a few licks, the animal receives a mild electric shock. Depending on the relative balance between the motivation to seek the drinking reward and facing the punishing shock, the rodent will stop or keep on drinking. The number of shocks the animal decides to stand, directly correlates with its approach behaviour. Another similar test, the *Geller-Seifter Conflict* Test (GSCT) (Geller *et al*., 1960) exploits a food-related reward instead of water. Although the human counterparts of the VCT and GSCT (described below) are less similar to the rodent variants than those of the OF and EPM, evidence suggests that these tests are equally valuable within cross-species approaches (Bach, 2021). Aupperle *et al*. (2011) developed a third-person view computer task, named *ACC conflict task*, in which human participants move an avatar on a runway to decide between their chances of receiving a conflict outcome (negative affective image/sound combined with point rewards) *v*. non-conflict outcome (affective image/sound coupled with no points). The trials were designed to elicit the simultaneous desire to approach the reward and avoid the negative affective punishment. A limitation of this paradigm is that the reward offered during the conflicting conditions can vary while the affective threat remains stable. As such, the task allows to study conflict-related neuronal activations that are associated with the higher salience of the reward, but not those that might be elicited by increased salience of the negative outcome. Within a similar rationale, two additional human-designed conflict tests, based on third-person view computer tasks, were developed by Bach *et al*. (2014). In these tasks, the player is instructed to press a key (Bach, 2015) or move on a rectangular grid (Bach *et al*., 2014) to collect virtual tokens under the threat of being caught by a virtual ‘predator’ and losing all previously collected tokens. Threat probability corresponds to the wake-up rate of the predator, and the magnitude of potential loss corresponds to the number of already collected tokens. The wake-up rate is signalled by different colours and tailored to result in 3 different wake-up probabilities if the player stays outside the safe place for 100 ms. The player cannot escape once the predator is active. When participants have to press the key, they tend to collect fewer tokens when the potential loss is higher (Bach, 2015). When participants have to move on the screen, they tend to explore and collect tokens early on, but as time progresses, the subjects retreat more to the safe place.

## The inhibitory role of the hippocampus in approach-related behaviours

In the ‘80 Jeffrey Gray and Neil McNaughton suggested that the mammalian hippocampus may represent a central component of the BIS, hence sustaining avoidance within the AAC (McNaughton and Gray, 2000; Gray and McNaughton, 2003). This interesting theory initially accounted for robust data showing how partial or total surgical hippocampus ablation in rodents leads to increased approach behaviours. Similarly, it was shown that local hippocampal infusion of anxiolytic drugs that inhibit excitatory hippocampal neurotransmission enhances approach-related actions (Gray and McNaughton, 2003). Lately, many studies described hippocampal functional polarisation, showing how the hippocampus is grossly divided into two portions, dorsal and ventral hippocampus (dHIP; vHIP) in rodents, respectively involved in spatial memory consolidation and affective/emotional processing (Kheirbek *et al*., 2013; Jimenez *et al*., 2018). Interestingly, anatomical segregation of hippocampal circuits has also been described in humans, being the anterior hippocampus homologous to the rodent vHIP and the posterior to the dHIP (Clark and Squire, 2013). Thus, these studies better address the vHIP in rodents and the anterior in humans as a relevant seat of emotional information processing possibly related to behavioural avoidance. In the following sections, we examine the latest experimental evidence, in particular optogenetic-mediated surgical circuitry characterisation in rodents, and fMRI in humans, supporting the role of the hippocampus in behavioural avoidance, and further endowing Gray's RST with spatial, molecular and metabolic determinants.

## Rodents optogenetic studies

Optogenetics refers to a biological technique to control the activity of genetically labelled neurons with light, an approach that significantly contributed in the last years to map brain functional connectivity (Adamantidis *et al*., 2015).

One of the first optogenetic evidence of hippocampal involvement in the approach-avoidance outcome showed that specific inhibition of glutamatergic neurons of the basolateral amygdala projecting to the vHIP promoted exploratory approach measured by the EPM test (Felix-Ortiz *et al*., 2013), while optogenetic activation of the same circuit limited exploration of the EPM open arms, increasing avoidance (Felix-Ortiz *et al*., 2013). Another interesting study outlined the role of an additional vHIP efferent pathway, directed to the medial PFC in the modulation of approach-avoidance (Padilla-Coreano *et al*., 2016). In particular, optogenetic inhibition of vHIP axon terminals projecting to the medial PFC biases the AAC towards approach behaviours measured as increased exploration in the EPM test, a profile that was further validated by the Novelty Suppressed Feeding test (Padilla-Coreano *et al*., 2016).

Recently, it was also described that optogenetic enhancement of the excitatory vHIP afferents that project to the lateral hypothalamus increases anxiety, shifting the AAC conflict toward exploratory avoidance as scored by the OF and EPM tests (Jimenez *et al*., 2018).

Unexpectedly, a similar positive optogenetic manipulation that was performed over those vHIP afferents that innervate basal amygdala (BA), limited fear memory encoding and retrieval in the contextual fear conditioning test but displayed no effect in OF test readouts (Jimenez *et al*., 2018). The authors concluded that positive manipulation of these two vHIP glutamatergic afferents affects different emotional domains in rodents. However, the inhibitory activity of the vHIP-BA circuit toward fear memory consolidation contrasts with the potential involvement of the hippocampus in behavioural avoidance. In general, the stronger the fear memory the less an animal will be engaged in approach behaviours. It is possible that, within the complexity of limbic circuitry, inner homoeostatic needs leave a minority of the hippocampal circuitry (including vHIP-BA) free to contribute to approach, while the majority, as reviewed here, contributes to behavioural avoidance; therefore, balanced functioning of these circuitries would serve to support adaptive behaviours. The aforementioned evidence suggests the hippocampus as a brain area involved in the discrimination of those advantages and potential threats that have to be weighted in decision making.

Chronic environmental stress including psychosocial trauma has been shown to modulate AAC towards avoidance in vulnerable mice (Toth and Neumann, 2013; Anacker *et al*., 2018). Thus, a question raises about whether the hippocampus contributes to translating stress into avoidance. In mice, chronic social defeat stress diminishes, in a subset of susceptible animals, the willingness to socially explore conspecific animals and exploratory behaviour. Such susceptibility is promptly reverted to resiliency by negatively regulating hippocampus excitability (Anacker *et al*., 2018). Interestingly, has also been shown that chronic treatment with imipramine, a tricyclic antidepressant drug increasing serotonin levels in the synaptic cleft, is able to restore a normal approach-avoidance balance in psychosocial stress susceptible mice previously evaluated as social avoidants (Tsankova *et al*., 2006). This effect is again mediated by an overall decrease of hippocampal excitability, in accordance with the inhibitory effect of the hippocampal serotonin receptors, 5HT1A, highly abundant in this area (Tsankova *et al*., 2006).

## Humans' studies

Recent functional magnetic resonance imaging (fMRI), and pharmacological and brain lesions studies confirm a relevant role for the hippocampus in the AAC in humans (Kheirbek *et al*., 2013; Bach *et al*., 2014; Weeden *et al*., 2015; Ito and Lee, 2016; Schumacher *et al*., 2018; Bach *et al*., 2019; Abivardi *et al*., 2020; Bryant and Barker, 2020; La-Vu *et al*., 2020; Yeates *et al*., 2020). For instance, Bach *et al*., conducted a fMRI study to investigate the role of the hippocampus in arbitrating ACC under various levels of potential threat, comparing neurologically healthy controls and patients with selective hippocampal lesions (Bach *et al*., 2014). Bold signal in the anterior hippocampus increased with the probability of predator attack, supporting the putative role of the hippocampus as a negative regulator of approach in AAC paradigms in humans. Importantly, the threat levels much less influenced the behaviour of patients with selective hippocampal lesions, which showed reduced passive avoidance behaviour and inhibition across all threat levels (Bach *et al*., 2014). The same AAC computer game was then used to investigate the impact of benzodiazepines and amygdala lesions on putative human anxiety-like behaviour (Korn *et al*., 2017). The task was administered to (i) a group of healthy controls after a single dose of lorazepam *v*. placebo and (ii) two patients with bilateral amygdala lesions *v*. age- and gender-matched healthy controls. Lorazepam and amygdala lesions reduced loss adaptation, decreasing patients' anxiety-related avoidance behaviour.

A more recent study from the same group (Bach *et al*., 2019) confirmed that, in humans, hippocampal lesions increase approach under conflict whereas amygdalar lesions impair the return to safety.

In summary, also human studies report a role of the hippocampus and amygdala in AAC under threat, linking the integrity of these regions to conditioned fear expression and inhibitory avoidance (Ito and Lee, 2016). Such new knowledge, however, warrants further inherent neuroimaging studies, based in particular on fMRI to better dissect inherent circuitry.

## Conclusion and future directions

Deepening the knowledge of AAC circuitry and mechanisms in rodents and humans holds a huge translational potential as it may help to unravel psychopathological elements of several psychiatric disorders featuring unbalanced AAC. Further studies combining hippocampus-focused functional brain imaging using the described AAC cross-species paradigms with clinical (i.e. questionnaire-based) evaluation of AAC and anxiety, should be performed to validate preliminary observation of increased hippocampal activity as a biomarker of threat or punishment sensitivity and avoidance, ultimately helping to refine psychiatric patient stratification and diagnosis along with treatment options and prognosis.

## Data Availability

All data used to write this paper is in the reference list.
