# Preclinical and clinical evidence on the approach-avoidance conflict evaluation as an integrative tool for psychopathology – CORRIGENDUM

**DOI:** 10.1017/S2045796022000816

**Published:** 2023-01-13

**Authors:** F. Rusconi, M. G. Rossetti, C. Forastieri, V. Tritto, M. Bellani, E. Battaglioli

**Affiliations:** 1 Department of Medical Biotechnology and Translational Medicine, Università degli Studi di Milano, Milano, Italy; 2 Department of Neurosciences and Mental Health, Fondazione IRCCS Ca’ Granda Ospedale Maggiore Policlinico, Milan, Italy; 3 Department of Neurosciences, Biomedicine and Movement Sciences, Section of Psychiatry, University of Verona, Verona, Italy

The last sentence on page 4 above the paragraph ‘Human studies’ of the article above includes a reference to ‘Tsankova, et al, 2006’ which was accredited to her by a mistake. The correct reference should read ‘*Yohn et al., 2020’:*

*‘This effect is again mediated by an overall decrease of hippocampal*

*excitability, in accordance with the inhibitory effect of*

*the hippocampal serotonin receptors, 5HT1A, highly abundant*

*in this area (Yohn et al)’.*

Both short and full reference to Yohn have been added in the article's PDF and XML accordingly:

*Yohn et al., 2020*

**Yohn CN, Dieterich A, Maita I, Bazer AS, Diethorn E, Ma D, Gergues MM, Hu P and Samuels BA**. (2020) Behavioral response to fluoxetine in both female and male mice is modulated by dentate gyrus granule cell activity. *Neurobiol Stress* **13**: 100257.

The authors apologise for the mistake.
